# Week 120 Efficacy of Tenofovir, Lamivudine and Lopinavir/r-Based Second-Line Antiretroviral Therapy in Treatment-Experienced HIV Patients

**DOI:** 10.1371/journal.pone.0120705

**Published:** 2015-03-30

**Authors:** Yang Han, Yijia Li, Jing Xie, Zhifeng Qiu, Yanling Li, Xiaojing Song, Ting Zhu, Taisheng Li

**Affiliations:** Department of Infectious Diseases, Peking Union Medical College Hospital, Beijing, China; McGill University AIDS Centre, CANADA

## Abstract

**Background:**

Tenofovir (TDF) and ritonavir-boosted lopinavir (LPV/r) were not introduced to China as second-line medications until 2009. The efficacy and safety of TDF/3TC/LPV/r based second-line regimen have not been evaluated in Chinese HIV patients who failed first-line regimens.

**Methods:**

This was a multicenter cohort study recruiting patients from Beijing, Shanghai, Guangdong, and Henan provinces between November 2008 and January 2010. Eighty HIV infected patients failing first-line regimens with serum creatinine lower than 1.5 times the upper limit of normal received TDF+ lamivudine (3TC)+ LPV/r were followed up for 120 weeks. CD4 cell count, viral load, and estimated glomerular filtration rate (eGFR) were monitored at each visit.

**Results:**

At baseline, 31.2% and 48.8% of patients had moderate/high-level resistance to TDF and 3TC, respectively; while 2.5% of patients had only low-level resistance to LPV/r. During 120 weeks of follow-up, virological suppression rate reached over 70% (<40 copies/ml) and 90% (<400 copies/ml), and median CD4 cell count increased from 157 cells/μL at baseline to 307 cells/μL at week 120. Baseline drug-resistance mutations had no impact on the efficacy of second-line antiretroviral therapy. Median eGFR dropped from 104.7 ml/min/1.73m^2^ at baseline to 95.6 ml/min/1.73m^2^ at week 24 and then recovered after week 96.

**Conclusion:**

This study for the first time demonstrated that TDF+ 3TC+ LPV/r was efficacious as second-line regimen with acceptable nephrotoxicity profiles in patients who failed zidovudine or stavudine based first-line regimens in China.

**Trial Registration:**

ClinicalTrials.gov NCT00872417

## Introduction

After being introduced to China in 2002, combination antiretroviral therapy (cART) effectively reduced mortality among adult HIV-infected patients in China [1]. At the initiation of the China National Free Antiretroviral Treatment Program, zidovudine (AZT), stavudine (d4T), didanosine (DDI) and lamivudine (3TC), plus nevirapine (NVP) or efavirenz (EFV) were used as first-line regimens. An inevitable issue associated with these regimens is treatment failure, with cumulative treatment failure rate reaching 50% after five years [1]. Second-line cART, which consists of tenofovir (TDF) and ritonavir-boosted lopinavir (LPV/r), was introduced in 2009. Its efficacy in Chinese adult HIV patients, however, has not been described.

TDF has been widely used in developed countries, though access to it is still limited in resource-limited areas, despite the fact that the World Health Organization (WHO) recommends TDF use[2] and that TDF might be more cost-effective even in these areas [3]. However, AZT and d4T are still being widely used in these areas [4]. Therefore, patients who have failed first-line therapy in resource-limited areas require relatively new regimens. TDF and protease inhibitors like LPV/r would be good candidates for second-line regimens.

Previously we reported a one-year observation of a prospective cohort consisting of treatment-experienced patients who received the second-line regimen. This study found that the TDF+LPV/r based regimen led to greater renal function decline compared with the control group, in which patients received AZT or d4T based regimens. However, this study did not evaluate the virological and immunological responses [5].

To this end, we present the findings of a 120-week prospective study. We evaluated the efficacy and safety of second-line cART regimens in first-line cART-experienced adult HIV patients.

## Methods

### Patients

Patients were recruited from Beijing, Shanghai, Guangdong, and Henan provinces between November 2008 and January 2010, as was described in our previous article [5]. Inclusion criteria were: (1) 18–65 years of age, (2) first-line cART failure (first-line cART was defined as AZT + 3TC + NVP/ EFV, d4T+3TC + NVP / EFV, AZT +DDI+ NVP / EFV or d4T + DDI + NVP / EFV) for at least one year with plasma HIV-1 viral load (VL) more than 400 copies per milliliter (copies/ml), (3) available estimated glomerular filtration rate (eGFR) and urinalysis results, and (4) stayed in this cohort for over 24 weeks. Exclusion criteria included: (1) previous use of TDF and/or protease inhibitor, (2) serum creatinine (SCr) >1.5 times the upper limit of normal (ULN). All recruited patients received second-line cART consisting of TDF+ 3TC+ LPV/r. The follow-up points included visits at 0, 4, 8, 12, 24, 36, 48, 72, 96, and 120 weeks, during which blood samples were collected for laboratory assessments including T cell subset and plasma VL.

### Measurements

Data collection was described in our previous report [5]. The CD4 cell count was determined by FACS-Canto (BD Biosciences, NJ, USA) using a standard technique according to the manufacturer's protocol. Separated plasma was immediately frozen at -80°C and transported to Peking Union Medical College Hospital’s central laboratory for viral load testing using the COBAS Ampliprep/TaqMan 48 real-time RT-PCR Test (Roche, CA, USA) according to the manufacturer’s instructions. The detection range was from 40 to 1,000,000 copies/mL.

Baseline drug resistance mutation was determined by using Pol sequence (described in our previous study [6, 7]) and by referring to the Stanford algorithm [8]. We also graded the levels of resistance (potential, low, medium, or high) by using the Stanford algorithm [8].

eGFR was calculated by the Chronic Kidney Disease Epidemiology Collaboration (CKD-EPI) formula, which is more accurate in estimating eGFR compared with other methods, especially when patients are on cART and [9] GFR is >60 mL/min/1.73m^2^ [10, 11].

### Ethics

All patients in this study signed informed consent. This research was approved by the Institutional Review Board of Peking Union Medical College Hospital. It was in compliance with relevant local laws and the principles expressed in the Declaration of Helsinki.

### Statistical analyses

All analyses reflected an intention-to-treat (ITT) model or per-protocol (PP) model and were performed using Stata 11.0. PP analyses included patients who finished 120 weeks of follow-up and missed less than four visits. All continuous values were summarized as medians with interquartile ranges (IQRs). Continuous variables were assessed with the Mann-Whitney test (unpaired comparison) or Wilcoxon matched-pairs sign-rank test (paired comparison, i.e. changes in CD4 cell count, eGFR), and categorical variables were assessed with chi-square test or Fisher’s exact test. Statistical significance was accepted at p< 0.05 for two-sided tests.

## Results

### Baseline characteristics

As summarized in Table 1 and S1 Fig, 80 treatment-experienced patients were included in this study. Most of them were male and were infected via blood transmission. Hypertension and diabetes were not prevalent in this group of patients. Regarding renal function, eGFRs in most patients were over 90 ml/min/1.73 m^2^, and only 4.9% of patients had proteinuria over 2+ in dipstick tests. Most patients were infected by HIV subtype B, and only seven (8.7%) patients were infected by non-B subtypes (six with CRF01_AE and one with CRF07_BC).

**Table 1:** Baseline characteristics (n = 80).

Age (years, IQR)	41 (38–47)
Male (n, %)	59 (73.8)
Route of transmission (n, %)	
MSM	7 (8.7)
Heterosexual	7 (8.7)
Blood	59 (73.9)
Others/Unknown	7 (8.7)
Hypertension (n, %)	9 (11.3)
Diabetes (n, %)	1 (1.3)
Body mass index (kg/m^2^, IQR)	21.7 (20.2–23.6)
CD4 cell count (cells/μl, IQR)	157 (69–272)
Viral load (log copies/ml, IQR)	4.35 (3.75–4.79)
eGFR (ml/min/1.73 m^2^, IQR)	104.7 (92.0–112.1)
Proteinuria (n, %)	
≥2+	4 (5.0)
<2+	71 (88.8)
Unknown	5 (6.2)
HBsAg status (n, %)	
Positive	2 (2.5)
Negative	75 (93.8)
Unknown	3 (3.7)
HCVAb status (n, %)	
Positive	56 (70.0)
Negative	21 (26.2)
Unknown	3 (3.8)
Subtype (n, %)	
Non-B	7 (8.7)
B	64 (80.0)
Unknown	9 (11.3)
DRM to TDF (n, %)	
N/P	45 (56.3)
L	10 (12.5)
M/H	25 (31.2)
DRM to 3TC (n, %)	
N/P	30 (37.5)
L	11 (13.7)
M/H	39 (48.8)
DRM to LPV/r (n, %)	
N/P	78 (97.5)
L	2 (2.5)

MSM, men who have sex with men; eGFR, estimated glomerular filtration rate; HBsAg, hepatitis B virus S antigen; HCVAb, hepatitis C virus antibody; DRM, drug resistance mutation; TDF, tenofovir; 3TC, lamivudine; LPV/r, ritonavir-boosted lopinavir.

As to baseline drug resistance profiles, almost half of the patients had resistance to TDF or 3TC before the initiation of second-line regimen (Table 1). Of note, moderate-to-high levels of resistance to 3TC were common. In contrast, resistance to LPV/r was low, with only two patients having low-level resistance mutations (2.5%). Drug resistance to non-nucleoside reverse-transcriptase inhibitors (NNRTI) was found in 66 patients (82.5%).

We also demonstrated transmitted drug resistance mutations in Table 2 (according to the World Health Organization 2009 List of Mutations for Surveillance of Transmitted Drug Resistant HIV Strains [12]).

**Table 2:** Transmitted drug resistants (TDRs) at baseline (n = 80).

	Total	Subtype B (n = 64)	Non-B (n = 7)	Unknown (n = 9)
NRTI TDR (n, %)				
M41L*	26 (32.5)	25 (39.1)	1 (14.3)	0 (0)
K65R	3 (3.8)	2 (3.1)	1 (14.3)	0 (0)
D67N/G/E	18 (22.5)	16 (25.0)	2 (28.6)	0 (0)
T69D	11 (13.8)	10 (15.6)	1 (14.3)	0 (0)
K70R/E	17 (21.3)	15 (23.4)	2 (28.6)	0 (0)
L74V/I	5 (6.3)	5 (7.8)	0 (0)	0 (0)
F77L	3 (3.8)	3 (4.7)	0 (0)	0 (0)
F116Y	2 (2.5)	2 (3.1)	0 (0)	0 (0)
Q151M	3 (3.8)	3 (4.7)	0 (0)	0 (0)
M184V/I	36 (45.0)	27 (42.2)	6 (85.7)	3 (33.3)
L210W	22 (27.5)	21 (32.8)	0 (0)	1 (11.1)
T215Y/F/I/S*	33 (41.3)	31 (48.4)	2 (28.6)	0 (0)
K219Q/E/N/R	21 (26.3)	19 (29.7)	2 (28.6)	0 (0)
NNRTI TDR (n, %)				
K101E	8 (10.0)	8 (12.5)	0 (0)	0 (0)
K103N/S*	28 (35.0)	27 (42.2)	1 (14.3)	0 (0)
V106M/A	4 (5.0)	4 (6.3)	0 (0)	0 (0)
Y181C/I/V	36 (45.0)	29 (45.3)	5 (71.4)	2 (22.2)
Y188C	2 (2.5)	2 (3.1)	0 (0)	0 (0)
G190A/S/E	22 (27.5)	17 (26.6)	4 (57.1)	1 (11.1)
P225H	2 (2.5)	2 (3.1)	0 (0)	0 (0)
PI TDR (n, %)				
V32I	1 (1.3)	1 (1.6)	0 (0)	0 (0)
V82F	2 (2.5)	1 (1.6)	1 (14.3)	0 (0)

NRTI, nucleoside reverse transcriptase inhibitor; NNRTI, non-nucleoside reverse transcriptase inhibitor; PI, protease inhibitor; TDR, transmitted drug resistant.

*, p<0.05

M184V/I, the major mutation accounting for resistance to 3TC, was found in 45% of patients. Of 25 (31.2%) patients with moderate-to-high levels of resistance to TDF, type 1 thymidine analog mutation (M41L and T215Y) was found in 21 patients. K65R, which was selected by d4T and ddI [13], was found in only three (3.8%) of patients. Positivity of Hepatitis B S antigen or antibody against Hepatitis C virus was not associated with higher rates of M184V/I, thymidine analog mutations or K65R mutations.

### Virological response

**Fig 1:**
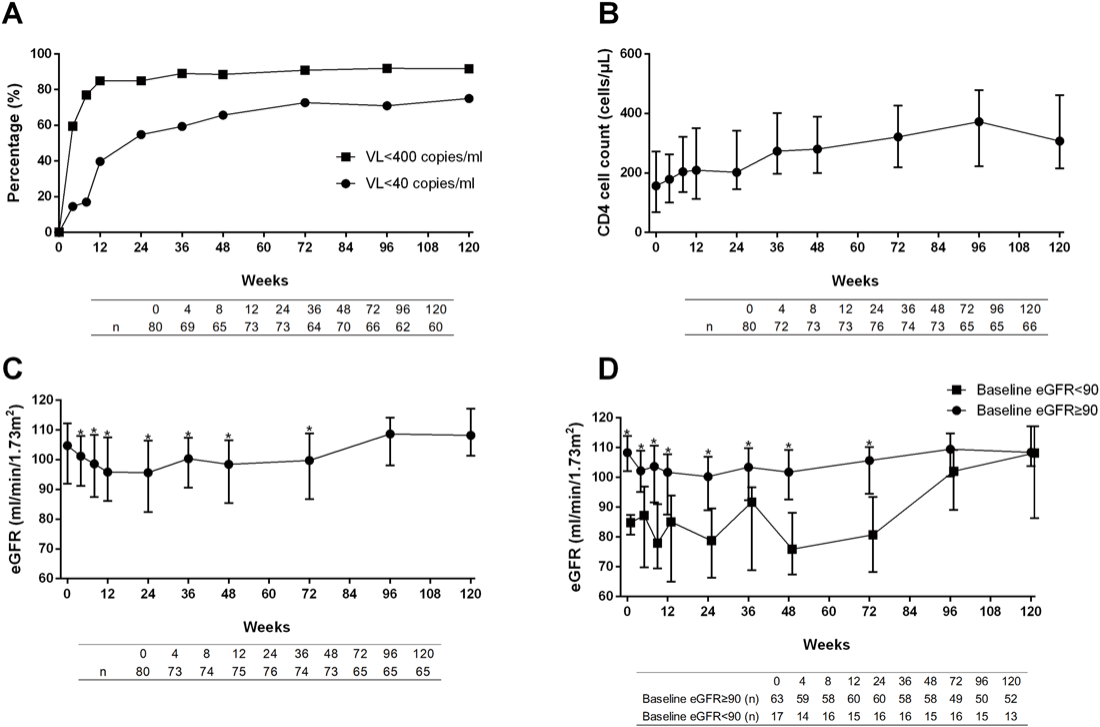
Efficacy and eGFR changes during 120-week treatment (ITT analyses). (A) Viral suppression rate (%) during 120-week follow-up. (B) CD4 cell count recovery during 120-week follow-up. (C) and (D) Estimated glomerular filtration rate (eGFR) during 120 weeks. Asterisks in (C) indicated significant differences compared with baseline levels. Asterisks in (D) indicated significant differences between groups with baseline eGFR over and lower than 90 ml/min/1.73m^2^.

During 120 weeks of follow-up, around 70% of patients reached viral suppression to VL<40 copies/ml in ITT analysis. In addition, almost 90% of patients achieved VL<400 copies/ml in ITT analysis (Fig 1A). PP analyses yielded similar results (S2A Fig). Baseline drug resistance was not associated with viral suppression. Viral suppression rates reached over 85% (<400 copies/ml) and 65% (<40 copies/ml) in different mutation groups, showing no statistical significance (S1 Table). We also evaluated the association of specific baseline drug resistance mutations (M41L, K65R, M184V/I, V32I, and V82F) and virological response and found that these specific mutations were not associated with virological response (at both <400 and <40 copies/ml levels, data not shown). The association of HIV subtype and virological response was not significant either (data not shown).

### Immunological response

Median CD4 cell count significantly increased from 157 cells/μL (IQR 69–272) at baseline to 307 cells/μL (IQR 216–461, p<0.001) at week 120 (Fig 1B). Median increase in CD4 cell count was 141 (IQR 60–252) cells/μL at week 120. PP analysis had similar results (S2B Fig). Baseline drug resistance profiles did not have an impact on CD4 cell recovery (S2 Table).

### Changes in renal function

After the initiation of this TDF-based second-line regimen, median eGFR gradually dropped from 104.7 ml/min/1.73m^2^ at baseline to 95.6 ml/min/1.73m^2^ at week 24. Median change in eGFR was -8.9 ml/min/1.73m^2^ at week 24 (IQR -15.7 to -2.1). It slowly increased but remained significantly lower than baseline level until week 96, when eGFR recovered to 108.6 ml/min/1.73m^2^, and remained at similar levels to those at baseline afterwards (Fig 1C). In order to further analyze the nephrotoxicity of this regimen, we also grouped our patients by their baseline eGFR (Fig 1D). PP analyses showed similar results with those in ITT analyses (S2C and S2D Fig).

## Discussion

To our knowledge, this is the first report in mainland China to demonstrate the efficacy and nephrotoxicity of TDF/3TC/LPV/r-based second-line cART.

At baseline, drug-resistance mutations were common in this cohort, since it mainly comprised of patients who initially received non-standard treatment and infrequent virological monitoring [1]. Given that 3TC was one of the medications in both first-line and second-line regimens, and adherence could not be guaranteed during the implementation of first-line therapy, resistance to 3TC was common. Resistance to TDF, on the other hand, could be due to inclusion of d4T and AZT in first-line regimen, which led to cross-resistance [14]. Resistance to LPV/r was rare, since LPV/r was not introduced to China until 2009, when this study was initiated.

As to virological response to this second-line regimen, around 70% and 90% of patients achieved viral suppression to <40 and <400 copies/ml, respectively. This result is similar to those of other studies with TDF and/or LPV/r based second-line regimens. In a Cambodian study with LPV/r as one of the second-line medications, viral suppression rate (VL<250 copies/ml) achieved 92.3% [15]. In fact, this viral suppression rate at week 48 (Fig 1A) approximates that of treatment-naïve patients receiving first-line cART in our previous studies, in which 58.8% of patients achieved VL <50 copies/ml and 82.9% achieved VL< 400 copies/ml [16]. However, in comparison with treatment-naïve patients receiving AZT+ 3TC+ NNRTI or d4T+ 3TC+ NNRTI, the viral suppression rate in this study is still lower [16, 17].

Notably, baseline drug-resistance profiles had no impact on virological responses (S1 Table). As most patients in our cohort harbored resistance to 3TC and TDF while only a small proportion of patients had LPV/r low-level resistance, we suggest that in this second-line regimen, LPV/r is crucial to viral suppression; in patients with resistance to TDF/3TC, this second-line regimen is nothing more than functional LPV/r monotherapy. As is known, PI has a higher genetic barrier compared to those of other classes of medications [18], and previous studies have demonstrated that PI monotherapy in patients with viral suppression might not be inferior to cART [19, 20]. However, a systemic review indicates that the overall efficacy of PI/r monotherapy is still inferior to that of cART in patients starting monotherapy after six months of viral suppression [21]. Unfortunately, there were no studies comparing LPV/r monotherapy with cART in patients with treatment failure. Despite this, we still argue that LPV/r is playing a pivotal role in this second-line regimen.

In terms of immunological response, patients in this cohort achieved CD4 cell count recovery similar to that in treatment-naïve patients receiving first-line therapy [17].

Although TDF has been reported to be associated with kidney injuries, in our cohort, eGFR slightly decreased at the beginning of this study and then slowly recovered to baseline levels. Interestingly, this slight decrease was only observed in patients with normal baseline eGFR levels; in patients with decreased baseline eGFR (median 84.8, IQR 80.7–87.4 ml/min/1.73m^2^), we only observed fluctuation after initiation of TDF and recovery after week 96. This is consistent with our previous one-year observation and other studies in both Asian and western countries, in which moderate decrease in eGFR was noted during 48 weeks of follow-up [5, 11, 22, 23]. However, we then observed recovery of eGFR, which is inconsistent with previous reports in both HIV-infected treatment-naïve patients and in healthy volunteers receiving pre-exposure prophylaxis. In these reports, eGFR kept decreasing at a slower rate after 48 weeks [11, 24–26]. This discrepancy could be due to the fact that most patients in our study had normal or only slightly impaired renal function before initiation of this regimen. In addition, most of them did not have risk factors for renal function impairment (i.e. diabetes, hypertension), except hepatitis C. Since HIV itself is an independent factor for renal impairment, and low CD4 cell count and high VL are associated with a higher risk of end-stage renal diseases [27], recovery of CD4 cell count and viral suppression by this second-line regimen might lead to recovery of renal function. We also speculate that, since patients in our study have experienced other nucleotide analogues, which have been demonstrated to have clinically similar renal toxicity profiles [26], our patients might have been “pre-conditioned” by previous NRTIs. In two previous 48-week studies with other NRTI-experienced patients, TDF was not associated with clinically significant changes in renal function [28, 29]. Unfortunately, there was no previous study with TDF-based second-line regimen to evaluate changes in renal function beyond 48 weeks, and further studies with larger sample size are warranted.

As to adherence, viral suppression <400 copies is a surrogate marker in our study. As seen in Fig 1A and S1 Fig A, viral suppression rate reached 90%, indicating good adherence in most patients.

The limitations to our studies are as follows. (1) The sample size is limited, and future studies with larger sample size are necessary to illustrate the efficacy of TDF/3TC/LPV/r based regimen in treatment-experienced patients. (2) Only patients with relatively good renal function were included in this study; therefore, it is difficult to evaluate the efficacy and safety profiles in patients with decreased eGFR. (3) Most patients in this cohort were infected via blood transmission, while in recent years, HIV infection via sexual transmission has become more common; therefore, the population characteristics might be deviated from the current situation.

In conclusion, this is the first study in mainland China to demonstrate the efficacy of TDF/3TC/LPV/r based second-line therapy in patients who failed first-line cART. Since access to TDF and/or LPV/r is still restricted in resource-limited countries, and the TDF/3TC/LPV/r based regimen is a good candidate for second-line cART, this study may also be meaningful for patients who failed first-line cART in these countries.

## Supporting Information

S1 Fig Enrollment flow chart. (TIF)

S2 Fig Efficacy and eGFR changes during 120-week treatment (PP analyses). (A) Viral suppression rate (%) during 120-week follow-up. (B) CD4 cell count recovery during 120-week follow-up. (C) and (D) Estimated glomerular filtration rate (eGFR) during 120 weeks. Asterisks in (C) indicated significant differences compared with baseline level. Asterisks in (D) indicated significant differences between groups with baseline eGFR over and lower than 90 ml/min/1.73m^2^.
(TIF)

S1 Table Viral suppression rate (%) and baseline resistance to second-line regimen. VL, viral load; N/P, no resistance or potential resistance; L, low-level resistance; M/H, moderate or high-level resistance; TDF, tenofovir; 3TC, lamivudine; LPV/r, ritonavir-boosted lopinavir. * Only two patients in our study harbored low-level resistance to LPV/r, one of whom lost to follow-up at week 84.
(DOCX)

S2 Table The association of baseline drug resistance mutation and median CD4 cell count and increase in CD4 cell count (median and interquartile ranges). VL, viral load; N/P, no resistance or potential resistance; L, low-level resistance; M/H, moderate or high-level resistance; TDF, tenofovir; 3TC, lamivudine; LPV/r, ritonavir-boosted lopinavir. * Only two patients in our study harbored low-level resistance to LPV/r, one of whom lost to follow-up at week 84.
(DOCX)
