# Supplementary figures and images for: Week 120 Efficacy of Tenofovir, Lamivudine and Lopinavir/r-Based Second-Line Antiretroviral Therapy in Treatment-Experienced HIV Patients
Source: PLoS One. 2015 Mar 30;10(3):e0120705. doi: 10.1371/journal.pone.0120705 (PMC4379083; doi:10.1371/journal.pone.0120705)

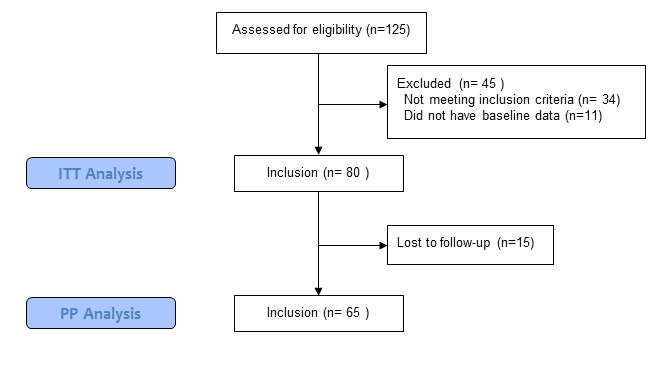

Supplement: S1 Fig — (TIF) [file pone.0120705.s001.tif]
