# Supplementary material for: Week 120 Efficacy of Tenofovir, Lamivudine and Lopinavir/r-Based Second-Line Antiretroviral Therapy in Treatment-Experienced HIV Patients
Source: PLoS One. 2015 Mar 30;10(3):e0120705. doi: 10.1371/journal.pone.0120705 (PMC4379083; doi:10.1371/journal.pone.0120705)
Supplement: S1 Table — VL, viral load; N/P, no resistance or potential resistance; L, low-level resistance; M/H, moderate or high-level resistance; TDF, tenofovir; 3TC, lamivudine; LPV/r, ritonavir-boosted lopinavir. * Only two patients in our study harbored low-level resistance to LPV/r, one of whom lost to follow-up at week 84. (DOCX) [file pone.0120705.s003.docx]

| S1 Table. Viral suppression rate (%) and baseline resistance to second-line regimen. | | | | | | | | | | |
| --- | --- | --- | --- | --- | --- | --- | --- | --- | --- | --- |
|  | VL<400 copies/ml | | | | | VL<40 copies/ml | | | | |
| Week | 0 | 24 | 48 | 96 | 120 | 0 | 24 | 48 | 96 | 120 |
| TDF resistance | | | | | | | | | | |
| N/P (%) | 0 | 85.7 | 89.7 | 93.9 | 90.0 | 0 | 64.3 | 64.1 | 66.7 | 70.0 |
| L (%) | 0 | 80.0 | 88.9 | 88.9 | 100.0 | 0 | 50.0 | 66.7 | 88.9 | 66.7 |
| M/H (%) | 0 | 85.7 | 86.4 | 90 | 90.5 | 0 | 38.1 | 68.2 | 70.0 | 85.7 |
| P value | NA | 0.899 | 0.873 | 0.688 | >0.999 | NA | 0.136 | 0.947 | 0.426 | 0.364 |
| 3TC resistance | | | | | | | | | | |
| N/P (%) | 0 | 82.1 | 88.9 | 90.9 | 87.0 | 0 | 57.1 | 66.7 | 68.2 | 73.9 |
| L (%) | 0 | 90.0 | 77.8 | 90.9 | 90.0 | 0 | 50.0 | 55.6 | 72.7 | 90.0 |
| M/H (%) | 0 | 85.7 | 91.2 | 93.1 | 96.3 | 0 | 54.3 | 67.7 | 72.4 | 70.4 |
| P value | NA | 0.907 | 0.505 | >0.999 | 0.465 | NA | 0.924 | 0.787 | 0.938 | 0.467 |
| LPV/r resistance | | | | | | | | | | |
| N/P (%) | 0 | 85.9 | 88.2 | 91.8 | 91.5 | 0 | 54.9 | 64.7 | 72.1 | 74.6 |
| L (%)* | 0 | 50.0 | 100.0 | 100.0 | 100.0 | 0 | 50.0 | 100.0 | 0 | 100.0 |
| P value | NA | 0.280 | >0.999 | >0.999 | >0.999 | NA | >0.999 | 0.543 | 0.290 | >0.999 |
| VL, viral load; N/P, no resistance or potential resistance; L, low-level resistance; M/H, moderate or high-level resistance; TDF, tenofovir; 3TC, lamivudine; LPV/r, ritonavir-boosted lopinavir.  * Only two patients in our study harbored low-level resistance to LPV/r, one of whom lost to follow-up at week 84. | | | | | | | | | | |
