# Supplementary material for: Week 120 Efficacy of Tenofovir, Lamivudine and Lopinavir/r-Based Second-Line Antiretroviral Therapy in Treatment-Experienced HIV Patients
Source: PLoS One. 2015 Mar 30;10(3):e0120705. doi: 10.1371/journal.pone.0120705 (PMC4379083; doi:10.1371/journal.pone.0120705)
Supplement: S2 Table — VL, viral load; N/P, no resistance or potential resistance; L, low-level resistance; M/H, moderate or high-level resistance; TDF, tenofovir; 3TC, lamivudine; LPV/r, ritonavir-boosted lopinavir. * Only two patients in our study harbored low-level resistance to LPV/r, one of whom lost to follow-up at week 84. (DOCX) [file pone.0120705.s004.docx]

| S2 Table. The association of baseline drug resistance mutation and median CD4 cell count and increase in CD4 cell count (median and interquartile ranges). | | | | | |
| --- | --- | --- | --- | --- | --- |
| Week | 0 | 48 | 96 | 120 | Increase in CD4 cell count at week 120 |
| TDF resistance | | | | | |
| N/P | 229 (91-299) | 329 (222-443) | 379 (282-593) | 305 (179-480) | 98 (26-249) |
| L | 187 (20-221) | 332 (236-437) | 418 (157-439) | 449 (253-657) | 217 (134-569) |
| M/H | 139 (64-184) | 235 (190-327) | 307 (210-417) | 292 (221-411) | 153 (91-214) |
| P value | 0.042 | 0.078 | 0.291 | 0.310 | 0.059 |
| 3TC resistance | | | | | |
| N/P | 188 (62-285) | 357 (212-470) | 394 (320-529) | 338 (168-517) | 141 (49-262) |
| L | 132 (32-141) | 212 (132-288) | 235 (201-407) | 254 (179-404) | 135 (74-218) |
| M/H | 186 (91-272) | 280 (211-370) | 372 (206-534) | 322 (235-462) | 147 (73-260) |
| P value | 0.160 | 0.069 | 0.163 | 0.349 | 0.831 |
| LPV/r resistance | | | | | |
| N/P | 163 (65-273) | 280 (207-392) | 378 (230-484) | 307 (219-462) | 143 (63-253) |
| L* | 119 (93-144) | 152 (118-186) | 199* | 151* | 58* |
| P value | 0.518 | 0.066 | NA | NA | NA |
| VL, viral load; N/P, no resistance or potential resistance; L, low-level resistance; M/H, moderate or high-level resistance; TDF, tenofovir; 3TC, lamivudine; LPV/r, ritonavir-boosted lopinavir.  * Only two patients in our study harbored low-level resistance to LPV/r, one of whom lost to follow-up at week 84. | | | | | |
